# “Antibiotic hardstop” on electronic prescribing: impact on antimicrobial stewardship initiatives in patients with community acquired pneumonia (CAP) and infective exacerbations of chronic obstructive pulmonary disease (IECOPD)

**DOI:** 10.1186/s12879-022-07117-8

**Published:** 2022-02-08

**Authors:** Semun Galimam, Brydon Panozzo, Kieran Muir, Ruchir Chavada

**Affiliations:** 1 Department of Pharmacy CCLHD, Wyong Hospital, Hamlyn Terrace, NSW Australia; 2 Division of Medicine CCLHD, Gosford Hospital, Gosford, NSW Australia; 3 Department of Microbiology and Infectious Diseases, NSW Health Pathology, Gosford, NSW Australia

**Keywords:** Antibiotic, Hardstop, Antimicrobial, Stewardship, Electronic

## Abstract

**Background:**

Antimicrobial resistance (AMR) remains a major public health threat and the exploration of interventions which may reduce inappropriate antimicrobial use are of particular interest. An Antibiotic Hardstop (AH) was included within the eMeds system introduced to the Central Coast Local Health District (CCLHD) in 2018. The function allows prescribers to set a predetermined time at which antibiotic orders would cease. By default, the function set prescribed length to 5 days with a view to encourage prescribers to review existing antimicrobial orders and reduce inappropriate use.

**Methods:**

Records of adult inpatients prescribed broad spectrum antimicrobials with a registered indication of community acquired pneumonia (CAP) or an infective exacerbation of chronic obstructive pulmonary disease (IECOPD) between the 1st of March 2017 and 31st May 2017 for the pre eMeds cohort and 1st March 2019 and 31st May 2019 for the post eMeds cohort were randomly selected from our local health network’s Guidance MS^®^ system. Baseline demographics, antimicrobial prescribing records and documented adverse events related to the AH function were collated/analysed. The days of therapy (DOT) and length of therapy (LOT) for each encounter were calculated manually and results analysed using a two-tailed t-test or Mann–Whitney U test.

**Results:**

Of patients eligible to have the AH function activated during their admission, 34% (n = 34) had the function deployed at least once. Following the introduction of eMeds mean DOT for the pooled indications cohort was reduced by 3.02 days (CI 95% 0.41–5.63, p < 0.05) and mean LOT by 1.97 days (CI 95% 0.39–3.55, p < 0.05). The hardstop function resulted in 2 cases of delayed or unintentionally ceased therapies.

**Conclusions:**

Following the introduction of electronic prescribing and AH, a significant reduction was observed in the DOT and LOT for antimicrobial use for inpatients with CAP and IECOPD without a significant increase in adverse events. Further research is required to determine the extent to which the AH functionality directly contributed to this effect and if the effect is present across a broader range of indications.

## Key points

- Local health district in regional NSW.
- Electronic prescribing introduced with Antibiotic Hardstop function.
- Defaulted to a 5-day therapy course.
- Significant reduction in antimicrobial use observed for CAP and COPD.
- No significant increase in adverse events.

## Introduction

Antimicrobial Resistance (AMR) poses an increasing threat to public health that necessitates urgent action. Around the globe a broad variety of strategies are being implemented in order to combat the issue [1]. Antimicrobial Stewardship (AMS) describes a system of activities and methodologies which aim to optimise antimicrobial use and maximise clinical outcomes while minimising unintended consequences, including the emergence of AMR [2]. A number of Information Technology (IT) tools have been utilised to facilitate AMS including computerised decision support systems, antimicrobial approval systems and surveillance systems [3]. The establishment of electronic medicine (eMeds) prescribing has opened up new opportunities to facilitate AMS [4]. The eMeds system in the Central Coast Local Health District (CCLHD) integrates patient information, medication charts, notes, clinical observations, pathology and imaging in a single electronic resource accessible from any computer with internet access with the aim of improving quality, safety and effectiveness of medication management. The use of eMeds with integrated AMS functionality has been associated with a variety of positive outcomes including increased adherence to prescribing guidelines and a reduction in antimicrobial prescribing overall. Software functions which have been integrated into eMeds systems to facilitate AMS have included antimicrobial hardstops (AH), prescribing alerts and specific indication order sets [5, 6].

There exists only a limited evidence base to guide optimal hardstop processes, with inconsistent conclusions regarding the optimal hardstop duration and overall impact of the intervention. Internationally, hospitals using an AH automated alert in EMR to identify patients that have received over 48 h of antimicrobial therapy have shown significantly decreased DOT of broad-spectrum antimicrobials [7, 8]. However, there is conflicting evidence within studies looking into the efficacy of EMR AH automated alerts in specific patient populations [9]. Furthermore, the large majority of the literature describing AH functions have been from the US, Europe and northern Asia with minimal studies describing AH interventions in an Australian population [10].

Community Acquired Pneumonia (CAP) and Infective Exacerbation of Chronic Obstructive Pulmonary Disease (IECOPD) both represent infective illnesses with a high disease burden and are indications for which broad-spectrum antimicrobials are commonly prescribed [11, 12]. Short course antibiotic therapy has demonstrated non-inferiority to longer courses of therapy for both indications and there is ongoing interest in understanding the impacts of short course antimicrobial therapy on clinical outcomes and antimicrobial resistance rates [13, 14]. The annual National Antimicrobial Prescribing Survey (NAPS) conducted in the CCLHD has consistently shown inappropriate antimicrobial use in the treatment of CAP and IECOPD due to prolonged duration of therapy. For these reasons, CAP and infective exacerbations of COPD were deemed suitable indications upon which to evaluate the effect of AH on antimicrobial prescribing behaviour.

## Methods

**Fig. 1:**
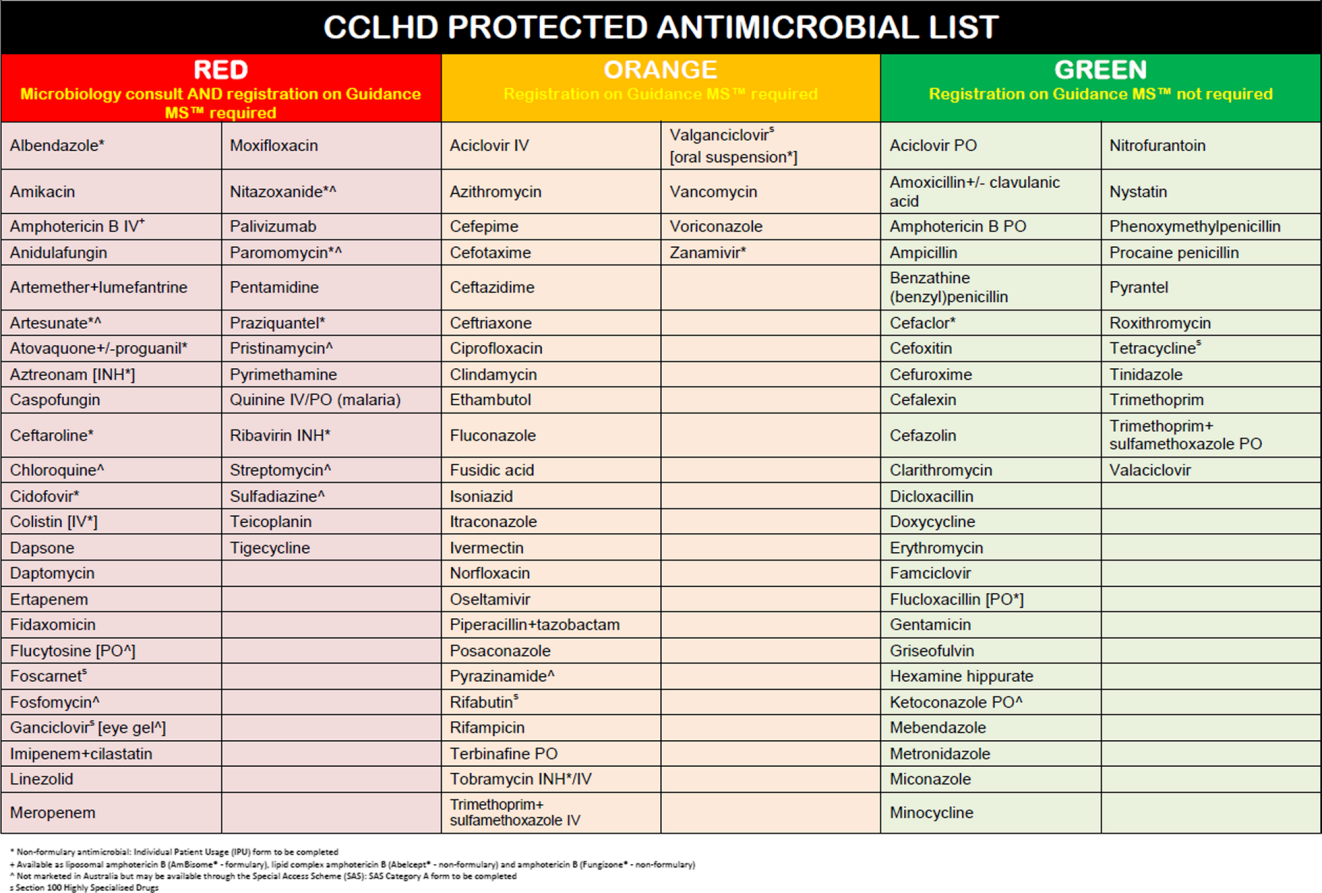
CCLHD Restricted Antimicrobials List

The Central Coast of NSW has 2 hospitals with 873 beds in total and services a population of approximately 350,000. The Central Coast Local Health District (CCLHD) introduced the AH function into eMeds in February/March 2018. The AH function allows prescribers to select a predetermined time at which the chosen medication order will cease unless the prescriber intervenes, or a new order is placed. Antimicrobials at CCLHD are grouped into a traffic light system (Fig. 1) with red encompassing antimicrobials which require consultation with and approval by an Infectious Diseases physician or clinical microbiologist as well as registration on our electronic decision support and approval system Guidance MS^®^ prior to prescribing. Orange antimicrobials require registration in our Guidance MS^®^ system and green antimicrobials are not routinely monitored. Red and orange antimicrobials represent either broad spectrum or high-risk antimicrobials. By default, antibiotic hardstops are set to 5 days, with prescribers having the option to disable this feature at the time of prescribing or during the course of the order.

The initial inclusion criteria compromised of patients who:

- Were 18 years old or older
- Were prescribed either a yellow or red restricted antimicrobial
- Received this antimicrobial within the 2 timeframes listed below

This study was designed as a pre-post interventional study. DOT and LOT for all antimicrobials (including non-restricted antimicrobials) were calculated and included in each patient’s final DOT and LOT tally. Initially a randomized selection of any patient (chronological lists of patients were generated for each time period with every 10th patient on the list selected) who satisfied the above selection criteria were included. However, upon statistical power analysis it was found that the only indications that had sufficient sample size to give the potential for finding a significant difference between groups of the data already collected were patients prescribed antimicrobials for CAP and IECOPD. One hundred records prior to the introduction of eMeds from the period 01/03/2017 to 31/05/2017 of adult inpatients prescribed broad spectrum antimicrobials with a registered indication of CAP or IECOPD were randomly selected from our Guidance MS^®^ system. The random selection process was repeated for the period 01/03/2019–31/5/2019 which followed the introduction of eMeds to CCLHD. Once all medical record numbers (MRN) and identifying information had been removed, baseline demographics, antimicrobial prescribing data and any documented adverse events related to the AH function were collated and analysed. Statistical analysis was performed using Stata Statistical Software: Release 16 (College Station, TX: StataCorp LP, 2019) with Days of Therapy (DOT) and Length of Therapy (LOT) calculated according to Public Health Ontario Antimicrobial Stewardship guidance [15].

Non-normal distribution was confirmed using Shapiro–Wilk tests for LOT and DOT subgroups (p = 0.00 for both measures), for pre eMeds and post eMeds subgroups (p = 0.00 for both measures) and CAP and IECOPD subgroups (p = 0.00 for both measures). The data naturally follows a non-normal distribution as the likelihood of a patient being on an antimicrobial is generally inversely related to the number of days a patient has been on the antimicrobial. LOT and DOT median and interquartile ranges were calculated using a two-sample t-test on the raw data. Due to the non-normal distribution of data and smaller sample sizes on subgroup analysis a two sample Mann–Whitney U test was used to confirm statistical significance.

## Results

The pre eMeds and post eMeds cohort were of comparable ages, comorbidities, rates of bacteraemia, and rates of involvement of an infectious diseases physician (Table 1). There was a small but statistically significant difference between the pre eMeds and post eMeds cohorts in the number of patients requiring ICU admission (20 vs 10 patients, p = 0.047) and patients from Residential Aged Care Facilities (RACF) (18 vs 30 patients, p = 0.047). A CURB-65 analysis of respiratory infection severity showed no significant difference between the pre eMeds group mean (SD) of 2.98 (1.21) and post eMeds group mean (SD) of 3.02 (1.18) (mean difference -0.04 severity rating, [95% CI, − 0.374–0.294], p = 0.814). Analysis showed no significant difference in infection severity between the CAP group mean (SD) of 2.98 (1.25) and IECOPD group mean (SD) of 2.06 (1.03) (mean difference − 0.077 severity rating, [95% CI, − 0.452—0.301], p = 0.689).

**Table 1:** Baseline characteristics of Pre eMeds and Post eMeds cohorts

	Pre eMeds	Post eMeds	p-value
Total patients	100	100	
Age in years, median (IQR)	74.5 (62–84)	77 (67–87)	0.1718
RACF	18	30	0.0469*
Bacteraemia	3	5	0.4705
CKD	10	19	0.0707
T2DM	17	28	0.0625
Respiratory disease	55	53	0.7766
PVD	2	5	0.2484
Immunosuppressed	7	13	0.1573
Haematological Malignancy	5	5	1.0000
ICU admission	20	10	0.0477*
Directed therapy	19	12	0.1714
ID involvement	21	14	0.1927
CURB65 mean (SD)	2.98 (1.21)	3.02 (1.18)	0.814
CURB65 categorised (%)			
0–1	12	10	0.6513
2	23	19	0.4874
≥ 3	65	71	0.3631

P-value calculated using two-sample test of proportions for categorical variables and two-sample t-test for continuous variables

Abbreviations: SD, standard deviation; CKD, chronic kidney disease; T2DM, type 2 diabetes mellitus; PVD, peripheral vascular disease; ICU, intensive care unit; ID, infectious diseases, RACF, Residential Aged Care Facility

^*^statistically significant p-value (p ≤ 0.05)

Following the introduction of electronic prescribing antimicrobial mean DOT was reduced by a mean 3.07 days (CI 95%, 0.47–5.67, p < 0.05). The mean LOT was reduced by 2.00 days (CI 95%, 0.42–3.57, p < 0.05). On subgroup analysis the reduction in DOT and LOT for the indication of CAP was not maintained, p = 0.123 and p = 0.145 respectively (Table 2). Additionally, no difference in the DOT or LOT for the indication of IECOPD was identified, p = 0.173 and p = 0.120 respectively. It is likely that this is secondary to underpowering upon stratification for subgroup analysis.

**Table 2:** Subgroup analysis Pre vs Post eMeds

	Pre eMEDS	Post eMEDS	
CAP			
Number of cases	64	84	
DOT (days)—mean	14.61	12	p = 0.123
LOT (days)—mean	8.78	7.31	p = 0.145
IECOPD			
Number of cases	35	17	
DOT (days)—mean	13.71	10.94	p = 0.173
LOT (days)—mean	7.77	5.64	p = 0.120

Multiple classes of antimicrobials were used in both patient cohorts. There was statistically significant lower rate of benzylpenicillin (0.074 vs 0.027, [95% CI, 0.014–0.081] p = 0.0076), ciprofloxacin (0.034 vs 0.010, [95% CI, 0.002–0.046], p = 0.043) and piperacillin-tazobactam (0.092 vs 0.272, [95% CI, 0.029–0.100], p = 0.001) use in the post eMeds cohort (Table 3). There was additionally a higher rate of ceftriaxone prescription in the post eMeds cohort (0.21 vs 0.29, [95% CI, -0.144—-0.010], p = 0.024).

**Table 3:** DOT and LOT by pre eMeds and post eMeds for top 5 most commonly used antimicrobials

Antibiotic	Pre eMeds		Post eMeds		
	Patients prescribed antibiotic	DOT (days)—mean	Patients prescribed antibiotic	DOT (days)—mean	Pre eMeds vs Post eMeds
Ceftriaxone	74	15.27	85	11.46	p = 0.009*
Doxycycline	57	17.05	49	12.14	p = 0.009*
Azithromycin	30	14.97	37	11.65	p = 0.130
Amoxicillin-Clavulanate	31	18.55	15	17.40	p = 0.763
Piperacillin-Tazobactam	32	15.906	8	12.625	p = 0.528

Antibiotic	Pre eMeds		Post eMeds		
	Patients prescribed antibiotic	LOT (days)—mean	Patients prescribed antibiotic	LOT (days)—mean	Pre eMeds vs Post eMeds
Ceftriaxone	74	8.77	85	6.54	p = 0.006*
Doxycycline	57	9.16	49	6.63	p = 0.021*
Azithromycin	30	8.23	37	6.78	p = 0.211
Amoxicillin-Clavulanate	31	10.77	15	10.73	p = 0.986
Piperacillin-Tazobactam	32	10.41	8	8.25	p = 0.498

P-value calculated using two-sample t-test for continuous variables

*statistically significant p-value (p ≤ 0.05)

34 of 100 post eMeds patients had the AH function activated at least once during their admission. Of those 34 patients, 10 had their antimicrobial continued by the team, 12 patients had their antimicrobial intentionally discontinued by the team (as documented in patient notes), 10 patients had their IV antimicrobials changed to PO. 2 patients had either a dose of antimicrobial missed/delayed, or their regimen unintentionally ceased.

## Discussion

The primary finding of this study was a reduction in overall DOT and LOT and can be reasonably hypothesized to produce a number of positive downstream effects. There is growing evidence to show that a reduction in antimicrobial use can result in a decrease in AMR, in particular within the hospital setting [16, 17]. Lower DOT/LOTs reflect reduced unnecessary use of antimicrobials and may lead to reduced antimicrobial resistance rates, reduced C. difficile infections and reduced infections due to MRSA, carbapenem resistant Pseudomonas aeruginosa and ESBL-producing enterobacterales [18]. It should be emphasized that the study did not find a significant difference in DOT and LOT specific to CAP and IECOPD individually but may be attributed to too small a sample size.

Of the 34 patients which received broad spectrum antimicrobials which triggered the AH, 22 (64.7%) of these either resulted in an intentional discontinuation of therapy or a de-escalation to oral antimicrobials. One meta-analysis has shown that AMS programs that look to prescribe according to guidelines (including recommended durations) and de-escalate therapy when appropriate have previously demonstrated a 35% relative risk reduction for mortality associated with guideline-adherent therapy and a 56% decrease in mortality associated with de-escalation of therapy [19].

The cost saving benefits of AMS intervention tools have been well described with AMS programs reducing antimicrobial costs by an average of 33.9% and length of stay by 8.9% [18]. The demonstrated reduction in DOT/LOTs directly reflects reduced antimicrobial use and could be attributed to the AH function prompting treating teams to consider ceasing antimicrobial therapy when indicated. Furthermore, intravenous antimicrobial therapy may often be the rate-limiting step preventing a patient’s discharge and the AH function prompting teams to consider an IV-to-oral change in antimicrobial therapy could be a contributing influence [20, 21]. Whilst it can be argued that many antimicrobials are comparatively inexpensive and a reduction in DOT/LOT may not make a sizeable difference in expenditure, many cost-saving benefits of AMS programs are found in indirect expense decreases (such as length of stay reduction, reduced side effect risk and cost of antimicrobial resistance) [18]. Direct cost savings in reduced antimicrobial use may be accounted for through specific pharmacy cost data analysis and may be an area for future investigation to account for all benefits.

**Fig. 2:**
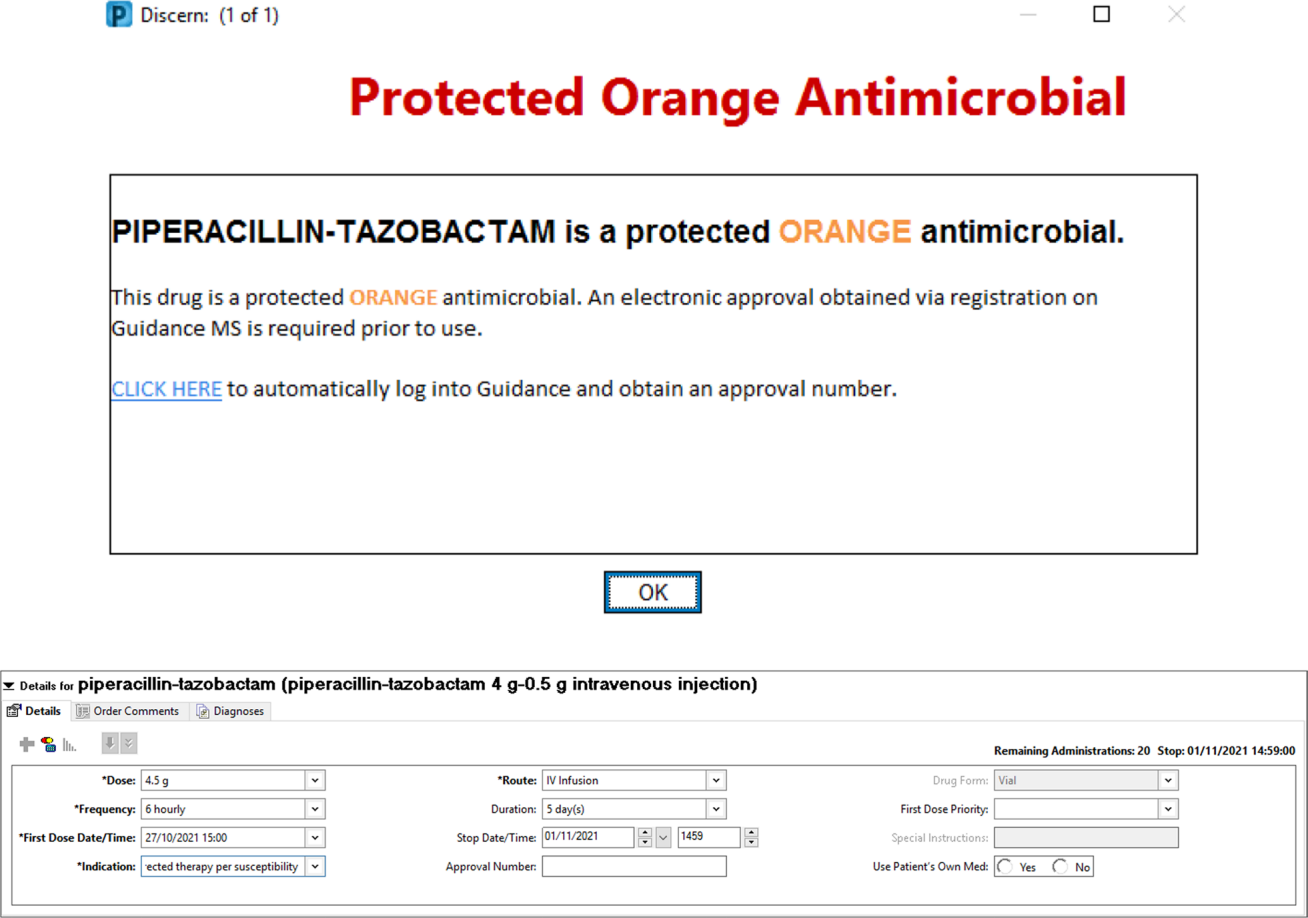
Guidance MS Restricted Antimicrobial Prescribing Alert and 5 day duration default

The default number of days an antimicrobial will be prescribed is automatically set to 5 days and unless treating teams are reviewing patient antimicrobials daily, then a patient may have their antimicrobials ceased when there is still an intention to treat. Contributors to this phenomena may include: time/resource constraints, an accidental oversight when reviewing a patient or teams not knowing that the AH function is utilized in CCLHD hospitals. While cessation of antimicrobials for patients who require them is a serious concern there have been no reported antibiotic hardstops which have resulted in any independently confirmed adverse outcomes for a patient in the CCLHD. Of the 100 patients from the post eMeds group, 2 patients had either a dose of antimicrobial missed/delayed, or their regimen unintentionally ceased. Of these 2 patients with missed/delayed doses or unintentionally ceased antimicrobial regimens, 1 patient missed 24 h’ worth of antimicrobials and was then changed to PO and 1 patient missed 48 h’ worth of antimicrobials and was then restarted on the same regimen with nil documented adverse outcomes. Contributing factors included the AH being activated on a weekend when there is reduced clinical staffing levels and an instance of eMeds being down and having to revert temporarily to paper charts. The outcomes and contributing factors of the two observed instances of patients having unintentional interruptions/discontinuations of therapy were reported to the CCLHD AMS Subcommittee and clinical governance units with subsequent remedial actions taken including ongoing medical officer education and clinical team awareness regarding the 5-day AH default (Fig. 2.) and antimicrobial prescribing restrictions (Fig. 1). Proposed mechanisms to address the potential risks include education during orientation for new clinical teams with intermittent reminders, an automated electronic task generated that prompts teams to review specific antimicrobials about to have the AH function employed (in particular if an AH is to be activated on a weekend), and regular auditing of Incident Information Management System (IIMS) reports regarding antibiotic hardstops.

Another potential negative impact of the hardstop functionality is so called “antibiotic hardstop fatigue”. The primary purpose of the AH function is to prompt teams to review the appropriateness of antimicrobials periodically. One concern is that if prescribing clinicians are required to repeatedly prescribe antimicrobials they may develop an automated response to repeatedly prescribing antimicrobials ceased by the AH function without assessing appropriateness. Alert fatigue is a recurring concern across multiple health professions that utilize patient electronic notes and charts with several studies looking into solutions [22, 23]. Suggested initiatives include regular education regarding the purpose of the AH function and its demonstrated benefits, adopting a tiered alert system giving greater prominence to antimicrobials involved in more serious infections, JMO feedback surveys at the end of each clinical rotation and antimicrobial stewardship teams to monitor for antimicrobials that may have been repeatedly prescribed without mindful review.

The study has a number of limitations some of which have been outlined above. No direct comparison has been made between the post eMeds cohorts that include and do not include an AH function. As such the effect of the electronic prescribing system on antimicrobial prescribing is difficult to differentiate from the effect of the AH function. It is unclear to what extent changes in general antimicrobial prescribing trends between 2017 and 2019 may have influenced DOT and LOT. Further investigation would be helpful in order to draw further conclusions regarding the demonstrated reduction in DOT and LOT. Patients on concurrent antimicrobials for indications other that CAP or IECOPD treatment were included in the final DOT and LOT count. Whilst this may potentially be considered an effect modifier, antimicrobials were counted in this manner for both pre and post AH cohorts and is unlikely to influence any statistical or practical inferences.

Patients were selected from a pool generated by the restricted antimicrobial software Guidance MS^®^. Antimicrobials are in general more likely to be restricted and require registering on Guidance MS^®^ if they are broad spectrum and higher risk. These antimicrobials are generally reserved for more severe or unusual infections and thus due to this selection method patients with mild CAP and/or IECOPD were not included. Inclusion of these patients may have revealed further insights into differences/similarities between the two groups. Adjusting the methodology to include patients prescribed non-restricted antimicrobials and those with mild CAP and IECOPD may be an area for future investigation.

There were several ongoing stewardship initiatives other than the hardstop function that may have influenced these results. During the time period the pre eMeds cohort was selected from, an audit analysing the rate of uptake of IV-to-PO switch recommendation made by the AMS team and an alignment with endorsed clinical guidelines analysis were taking place. During the time period the post-eMEDS cohort was selected from, an antibiotic allergy assessment/delabelling audit and vancomycin prescribing audit were taking place. Whilst the authors believe that these may have influenced patterns of antimicrobial prescribing directly through day-to-day AMS ward rounds during the audits and indirectly through diverting resources away from clinical work towards quality improvement projects, we do not believe that this would have significantly affected results given these were the data collection and analysis phases of each initiative and feedback/action from the pre-eMEDS cohort initiatives had not occurred by the time of the post-eMEDS cohort time period due to changes in AMS resources.

The small number of patients had either a dose of antimicrobial missed/delayed, or their regimen unintentionally ceased, and the lack of adverse clinical outcomes due to these missed/delayed/ceased doses made it difficult to assess the severity of potential consequences in patients who do not receive antimicrobial therapy due to the hardstop function. Whilst it could be speculated that giving antimicrobials for a shorter duration than intended for CAP and IECOPD could lead to undertreated infection (and costs associated with increased care needs and lengths of hospitalization) studies have shown that short-course antibiotic therapy of 3–5 days (i.e., shorter duration than the default time until hardstop activation) may be equally effective to longer traditional courses of therapy [13, 14]. Associations between intentional hardstop activation and any adverse outcomes however is an important consideration and an area for further exploration.

The small sample size when stratifying by either IECOPD or CAP (in particular for IECOPD with 35 pre-eMEDS patients and 18 post-eMEDS patients) meant that it was unlikely this study was to elicit significant differences in DOT/LOT between cohorts for specific indication. Whilst trends were observed, neither CAP nor IECOPD found a significant difference in DOT/LOT individually. This can also be said when looking at DOT and LOT stratified by the 5 most used antimicrobials with the statistically significant reduction in DOT and LOT observed in the two most commonly used antimicrobials but not for the others (Table 3). Further studies utilizing the capabilities of electronic medication management and data extraction may be able to account for this small sample size and may warrant further investigation.

## Conclusion

The electronic prescribing landscape remains ripe with opportunities to integrate AMS tools in an effort to further improve the behaviour of antimicrobial prescribers. It also opens up opportunities for future research, including the ability to pool and analyse large amounts of patient data to help reveal concepts otherwise hidden by the restrictions of labour intensive manual methods. The observed reductions in DOT and LOT in patients with CAP and IECOPD are clearly suggestive that the AH function has exerted a strong influence on antibiotic prescribing behaviour. It prompts the prescriber to evaluate the patients’ current clinical condition and consider consulting therapeutic guidelines in order to examine current practice. The results from this study demonstrate the potential role that AH functionality may have in reducing inappropriate antimicrobial use and improving the culture of antibiotic prescribing in the inpatient environment and gives justification for further exploration. There remain a number of challenges including demonstrating broad efficacy of the intervention across a range of indications and ensuring that AMS programs aimed at improving antimicrobial prescribing do not adversely affect patient care.

## Data Availability

The data that support the findings of this study are available from Central Coast Research Governance Office but restrictions apply to the availability of these data, which were used under license for the current study, and so are not publicly available. Data are however available from the authors upon reasonable request and with permission of Central Coast Research Governance Office.
